# A Hybrid Explainable AI Framework (HXAI) for Accurate and Interpretable Diagnosis of Alzheimer’s Disease

**DOI:** 10.3390/diagnostics15243118

**Published:** 2025-12-08

**Authors:** Fatima Hasan Al-bakri, Wan Mohd Yaakob Wan Bejuri, Mohamed Nasser Al-Andoli, Raja Rina Raja Ikram, Hui Min Khor, Mohd Syafiq Mispan, Norhazwani Md Yunos, Noor Fazilla Abd Yusof, Muhammad Hafidz Fazli Md Fauadi, Abdul Syukor Mohamad Jaya, Nor Aiza Moketar, Noorrezam Yusop, Kharismi Burhanudin, Tyanita Puti Marindah, Anugrayani Bustamin, Zahir Zainuddin, Deasy Wahyuni, Umi Kalsom Ariffin

**Affiliations:** 1 Faculty of Information and Communication Technology, Universiti Teknikal Malaysia Melaka, Melaka 76100, Malaysia; forignn@gmail.com (F.H.A.-b.); raja.rina@utem.edu.my (R.R.R.I.); nor.aiza@utem.edu.my (N.A.M.); noorrezam@utem.edu.my (N.Y.); 2 Faculty of Artificial Intelligence and Cyber Security, Universiti Teknikal Malaysia Melaka, Melaka 76100, Malaysia; wanie@utem.edu.my (N.M.Y.); elle@utem.edu.my (N.F.A.Y.); hafidz@utem.edu.my (M.H.F.M.F.); syukor@utem.edu.my (A.S.M.J.); kharismi@utem.edu.my (K.B.); 3 Faculty of Computing Informatics, Multimedia University, Cyberjaya 63100, Malaysia; 4 Department of Medicine, Faculty of Medicine, University of Malaya, Kuala Lumpur 50603, Malaysia; hmkhor@um.edu.my; 5 Fakulti Teknologi dan Kejuruteraan Elektronik dan Komputer, Universiti Teknikal Malaysia Melaka, Melaka 76100, Malaysia; syafiq.mispan@utem.edu.my; 6 Faculty of Engineering, Universitas Hasanuddin, Gowa 92171, Indonesia; tyanitaputi@unhas.ac.id (T.P.M.); anugrayani@unhas.ac.id (A.B.); zahir@unhas.ac.id (Z.Z.); 7 Fakultas Ilmu Komputer, Universitas Dumai, Riau 28811, Indonesia; deasywahyuni1@gmail.com; 8 Lee Kong Chian Faculty of Engineering and Science, Universiti Tunku Abdul Rahman, Kajang 43000, Malaysia; madamumikalsom2025@gmail.com

**Keywords:** Alzheimer’s disease, explainable AI, ensemble learning, SHAP, Grad-CAM

## Abstract

**Background/Objectives**: In clinical practice, Explainable AI (XAI) enables non-specialists and general practitioners to make precise diagnoses. Current XAI approaches are limited, as many rely solely on either presenting explanations of clinical data or presenting explanations of MRI, or presenting explanations in unclear ways, reducing their clinical utility. **Methods**: In this paper, we propose a novel Hybrid Explainable AI (HXAI) framework. This framework uniquely integrates both model-agnostic (SHAP) and model-specific (Grad-CAM) explanation methods within a unified structure for the diagnosis of Alzheimer’s disease. The dual-layer explainability constitutes the main originality of this study, as it provides the possibility of interpreting quantitative (at the feature level) and spatial (at the region level) data within a single diagnostic framework. Clinical features (e.g., Mini-Mental State Examination (MMSE), normalized Whole Brain Volume (nWBV), Socioeconomic Status (SES), age) are combined with MRI-derived features extracted via ResNet50, and these features are integrated using ensemble learning with a logistic regression meta-model. **Results**: The corresponding validation reflects the explainability accuracy of these feature-based explanations, with removal-based tests achieving 83.61% explainability accuracy, confirming the importance of these features. Model-specific information was used to explain MRI predictions, achieving 58.16% explainability accuracy of visual explanations. **Conclusions**: Our HXAI framework integrates both model-agnostic and model-specific approaches in a structured manner, supported by quantitative metrics. This dual-layer interpretability enhances transparency, improves explainability accuracy, and provides an accurate and interpretable framework for AD diagnosis, bridging the gap between model accuracy and clinical trust.

## 1. Introduction

Accurate and early diagnosis of Alzheimer’s disease (AD) is critical for effective management and treatment of the disease [1], but it remains a challenge due to the complex presentation of the condition and its overlap with other forms of dementia [2,3]. Enlargement of the ventricles is one of the oldest and most prominent signs of AD [4]. Artificial Intelligence (AI) plays a major role in the evolving digital age, impacting various aspects of human interaction [5]. Leveraging deep learning techniques, such as convolutional neural networks (CNNs), is crucial to addressing these challenges [6]. Recent advances in AI, particularly in the fields of machine learning and deep learning, offer promising solutions to enhance the accuracy and efficiency of AD diagnosis. These techniques can analyze complex imaging and clinical data to achieve high diagnostic accuracy. However, its clinical prevalence is hampered by its “black box” nature. Traditional AI is difficult to trust in critical applications [7,8,9].

Clinicians cannot easily understand the reasoning behind an AI’s decision, leading to a crisis of trust and accountability in high-stakes medical environments. This critical trust gap is what Explainable AI (XAI) aims to bridge. XAI explains AI decision-making, making it transparent and explainable. However, current XAI, especially in the context of AD diagnosis, are still considered black boxes that reduce trust in the use of AI within clinical environments. In Alzheimer’s research, many existing studies have relied solely on MRI data or only on clinical data [10]. However, a reliable diagnosis of AD requires both clinical assessments and MRI, and neglecting this integration limits diagnostic explainability and reduces confidence in AI-based tools for diagnosis.

This study aims to bridge the gap in AI, which is often viewed as “black boxes” with limited transparency and limited decision-making. This is where XAI comes in, providing explanations for AI decision-making in the context of AD.

Most current XAI technologies suffer from limitations in compatibility with clinical practice, providing complex explanations or ones that are not linked to underlying medical knowledge. Some frameworks rely on highlighting irrelevant areas in MRI images, which reduces doctors’ confidence in their results. Therefore, this study aims to improve the accuracy of the framework and enhance the understandability of its output by integrating clinical data with key anatomical regions of the brain. This integration of explanations ensures the transparency of the framework and its ease of application in busy clinical settings. The existing techniques that are applied to MRI, which, although powerful, often highlight irrelevant brain regions or lack contextual explanations, thus limiting their clinical value [11].

This paper demonstrates how SHAP (rule-based) and Grad-CAM (visualization-based) can be combined in a single framework. SHAP is a model-agnostic, rule-based technique that quantifies the contribution of each clinical feature to the model’s prediction, providing a numerical explanation at the feature level. Grad-CAM, on the other hand, is a model-specific visualization-based method that generates heat maps to highlight important regions in MRI scans, showing which areas of the brain most strongly influenced the model’s decisions. Instead of relying solely on numerical explanation or solely on visual explanation, the proposed approach integrates both to provide a more comprehensive, clinically relevant, and interpretable output. Our previous work [12] discussed the use of mid-slice MRI scans that clearly show the lateral ventricles, instead of analyzing full MRI sequences. While the hippocampus is more specific in diagnosing AD, this study focuses on mid-slice MRI of the lateral ventricles because it clearly shows ventricular hypertrophy, which is a prominent and easily identifiable structural change associated with AD. The use of ventricular images provides sufficient discriminatory information for classification, while simplifying the visualization process for Grad-CAM, and ensuring that the resulting heat maps are interpretable and clinically useful. Building on this, the present study integrates this imaging strategy into a Hybrid Explainable AI Framework (HXAI), combining SHAP and Grad-CAM to address the black-box problem in AI-based AD diagnosis.

The proposed HXAI framework is designed as a unified diagnostic system that integrates SHAP for rules-based clinical interpretations and Grad-CAM for MRI interpretations through a structured bilayer architecture. The process begins with a pre-trained group model that produces the initial classification of AD/MCI/CN using clinical features and selected mid-slice MRI scans. Its outputs are then passed to two successive interpretation units, SHAP, which generates clinical feature significance scores, and Grad-CAM, which produces heat maps highlighting the lateral ventricles in mid-slice MRI. This approach tackles one of the major challenges in medical AI: while models may achieve high accuracy, clinicians often struggle to understand how decisions are made.

According to a comprehensive review by [13], XAI encompasses both AI systems and the explanations they provide, aiming to create models that are not only accurate but also interpretable and trustworthy. XAI is a field that focuses on making AI models’ decisions understandable and interpretable by humans [14]. The decision-making capabilities of these systems are categorized into two approaches: (1) relying solely on learned features and (2) combining learned features with additional clinically relevant information, such as the size of the lateral ventricles. Both strategies can yield explainable outcomes through XAI frameworks. However, many of these models still lack robust explainability, making them less practical for clinical decision-making despite their high accuracy. The importance of explainable AI in medical imaging lies in the following key areas:

• Transparency: allowing clinicians to understand the reasoning behind AI decisions, thereby enhancing trust in technology.
• Clinical usability: supporting non-specialists and general practitioners in effectively interpreting AI outputs.
• Verification: ensuring AI decisions align with clinical knowledge, such as focusing on specific brain regions in MRI to detect AD.
• Improving patient outcomes: enabling earlier and more accurate diagnoses by making AI outputs both actionable and interpretable.

### Specific Contributions and Clinical Relevance

Despite the recognition of XAI, current approaches to diagnosing AD often fall short of meeting the overall needs of clinical practice. This work specifically addresses three critical gaps in the current literature:

1. Many studies offer interpretations based solely on clinical data or MRI scans, failing to reflect the integrated diagnostic process used by clinicians to diagnose AD. This work bridges this gap by proposing a hybrid framework that seamlessly integrates interpretations of both data types, providing a unified and comprehensive diagnostic basis.
2. XAI techniques, when applied to MRI, often highlight visually striking but clinically irrelevant areas, which undermines clinical confidence. Our framework directly addresses this gap by focusing exclusively on the lateral ventricles. Our conceptions of Grad-CAM are inherently consistent with clinical knowledge, ensuring that the highlighted regions are medically significant.
3. Previous work often presents SHAP diagrams or Grad-CAM thermal maps as endpoints, without integrating them into a coherent clinical narrative. This study presents a structured approach to explainability.

Therefore, the primary scientific contribution of this work directly addresses a key clinical need: providing transparent, multimodal, clinically grounded justifications that enhance physician confidence and improve the practical usability of AI-assisted Alzheimer’s diagnosis for both general practitioners and non-specialists in resource-conscious healthcare settings.

## 2. Related Work

As AI models become more complex, concerns have grown among researchers and clinicians about the “black box” nature of these algorithms and the need to understand their decision-making processes. Techniques such as PDA and PGM have been developed to improve the explainability of models [15], but they are still not understood by the end user. XAI techniques, which bring transparency and explanation to AI models in clinical settings, have become invaluable for identifying AD.

### 2.1. Current XAI Techniques Based on the Applicability of Models in Medical

#### 2.1.1. Model-Agnostic

• Shapley Additive Explanations (SHAP)

SHAP is a widely used XAI technique that offers model-agnostic explanations by breaking down individual diagnosis results into contributions from each feature. In AD diagnosis, SHAP has been applied to highlight specific features that are crucial in forecasting disease onset or progression using MRI images, as seen in studies by [16,17,18,19]. Although SHAP aids in interpreting deep learning models by showing which features influence diagnosis outcomes, it often remains a “black box,” providing limited insight into the interactions among features, as noted in studies like [20,21,22].

In practice, SHAP can be used on a model trained on brain MRI data to assign importance scores to features, though it may not always deliver an intuitive view of how these features combine to inform the overall diagnosis [17]. According to [17], the Shapley value of a feature quantifies its impact on the model’s output, computed by weighing and summing over all combinations of feature values:

(1) $$\phi_{j}\left(val\right)=\sum_{\left\{S\subseteq \left\{1,\ldots ,p\right\}\setminus \left\{j\right\}\right\}}\frac{\left|S\right|!\left(p-\left|S\right|-1\right)!}{p!}\left(val\left(s\cup \left\{j\right\}\right)-val\left(s\right)\right)$$

where *S* is a subset of features used by the model, *p* is the total number of features, and *x* represents the feature vector for the instance in question. The function *valₓ(S)* reflects the model’s output for the features in *S*, prioritized over those outside *S*:

(2) $${val}_{x\left(S\right)}=\int \hat{f}\left(x^{1},\ldots ,x_{p}\right){dP}_{\left\{xₛ=S\right\}}-E_{X\left(\hat{f}\left(X\right)\right)}$$

The Shapley value uniquely satisfies the principles of efficiency, symmetry, nullity, and additivity, all essential for fair attribution. As a result, a feature’s Shapley value reflects its contribution to the deviation between the model’s diagnosis outcome and the average diagnosis result, capturing the feature’s impact in the context of the specific case.

• Local Interpretable Model-Agnostic Explanations (LIME)

LIME is another versatile and model-agnostic AI technique that provides explanations for individual diagnosis outcomes by approximating the AI model’s behavior locally around a specific instance. In AD diagnosis, for example, LIME can be used to clarify why a brain MRI scan was flagged as indicative of the disease by identifying the most influential features in that decision. Studies such as [22,23] demonstrate how LIME reveals these feature impacts by altering the input data and observing shifts in the model’s output.

Despite its limitations, LIME remains a practical tool for bringing explainability to complex AI models. As highlighted in [23,24] LIME occasionally identifies areas outside the brain as important in decision-making, raising questions about the validity of such explanations. According to [25] the core of LIME’s approach lies in the following optimization equation, which helps determine the optimal interpretable model:

(3) $$\epsilon \left(x\right)=argg\in Gmin\left[L\left(f,g,\pi x\right)+\Omega \left(g\right)\right]$$

Here, *L*(*f*, *g*, *πₓ*) represents the local fidelity loss function between the black-box model *f* and the interpretable model *g* in the neighborhood *πₓ* and Ω(*g*) measures the complexity of the explanation model.

#### 2.1.2. Model-Specific

• Gradient-weighted Class Activation Mapping (Grad-CAM)

Grad-CAM is a model-specific XAI technique. Because it relies on internal model structures, it is widely used to interpret decisions of deep learning models. Grad-CAM highlights the regions of the input image that have the greatest impact on the model’s predictions. Grad-CAM can identify the brain regions most relevant to the model’s diagnosis. It also works by using gradients flowing backward from the model’s output layer to identify the features in the input that contributed most to the prediction.

A deep learning framework was developed at [26] to identify and interpret AD stages using artificial intelligence techniques. This framework analyzes different brain sections and uses Grad-CAM to visualize the regions identified by the AlzheimerTriMatterNet model. It was observed that models sometimes overemphasize regions outside the brain, reducing the clinical explainability of the results.

Grad-CAM visualizations provide insight into the features utilized by models to detect AD. However, it has been observed that the models occasionally highlight regions outside the brain, which may reduce the clinical explainability of the results.

In another study [27] the Grad-CAM-based XAI framework was applied to MRI images. A comprehensive evaluation was conducted on CNN models trained on real and synthetic MRI images, with gradients extracted from the final convolutional layer. However, the resulting explanations were unclear or difficult to interpret effectively.

• Layer-wise Relevance Propagation (LRP)

LRP is an XAI technique used to interpret diagnosis results by assigning relevance scores to each input feature. LRP creates heat maps in the input space, highlighting the importance of each voxel contributing to the final classification result. This method identifies the most important features, enhancing the explainability of complex neural networks [28].

In the study by [29] CNN was trained to distinguish between Alzheimer’s patients and healthy controls (HC) using structural MRI data. The researchers proposed using LRP to visualize the decision-making process in a CNN for differential classification based on MRI data. However, while these heatmaps show which regions influenced the model’s decision, they do not provide information about the underlying reasons behind the model’s focus on these regions. This limitation makes the explanations less interpretable and sometimes difficult to understand for end users, especially in clinical contexts.

### 2.2. Incorporating Domain-Specific Explanations

The general explanations provided by traditional XAI techniques often fail to fit the specific requirements of medical diagnostics, which are critical indicators of AD progression. Without these features, explanations lack consistency with clinical knowledge, limiting their utility in decision-making. For AD diagnosis, explanations must be rooted in medical knowledge, ensuring their relevance to clinical practice. The author [30] focused on the use of DTI data and machine learning techniques to classify Alzheimer’s patients and white matter characteristics as potential biomarkers of AD. The article [31] presented promising results using the hippocampus and VGG16 model with transfer learning for the diagnosis of AD. However, these and similar approaches often prioritize model accuracy over the clinical applicability of the interpretations they produce. Clinically validated XAI should not only point to the important features but should also place them in the context of their importance within the interpretation of the known pathophysiology of AD, which is often missing. The article [25] concludes that an XAI framework should provide clear and understandable explanations for why a model arrives at specific diagnosis outcomes and guide clinicians in making informed decisions about patient care.

#### Strengths and Weaknesses of Existing Approaches

Balancing high accuracy with explainability in models that detect AD is a key challenge in developing AI models for clinical applications.

Many existing methods prioritize accuracy, such as [32,33,34], these models are strong at achieving high results but weak at explainability. This high performance typically comes at the expense of explainability, so they are not widely used in clinical applications. For example, models such as CNN [35,36] can detect subtle patterns indicative of AD. However, their complex architecture creates a “black box,” and this lack of transparency limits trust and adoption in clinical settings. Healthcare decisions require clear, interpretable reasoning, not a simple yes or no answer.

On the other hand, XAI techniques such as SHAP [16,17] aim to fill this gap by providing post hoc explanations of model predictions, but they fail to deliver a fundamental understanding to the end user. This makes the need for innovative approaches that balance accuracy and explainability imperative, ensuring the explainability of AI tools and their potential use in clinical practice. The following table compares previous work across different databases and data types for AD diagnosis and decision explanation. All articles studied are still incomplete due to the imbalance between high accuracy and explainability. Table 1 provides an analysis of the trade-offs between performance and interpretability in previous AD detection studies. It demonstrates that current approaches are often limited to a single explanatory model, either independent of or specific to a given model, leading to incomplete interpretations and undermining clinical confidence. This analysis highlights the specific research gap that this thesis aims to address.

## 3. Problem Formulation

The fundamental problem addressed by this study is the lack of clinically meaningful and multimodal interpretability in current AI systems for AD diagnosis. Current diagnostic models often rely entirely on visual interpretations based on MRI, which may highlight anatomically or non-cerebral regions, as reported in several XAI studies [21,23,38], or rely exclusively on numerical feature significance scores derived from clinical data, providing an incomplete justification that clinicians cannot reliably interpret [39,40]. In addition, while CNN-based architectures excel at feature extraction and classification [41,42,43,44], they often behave as “black boxes,” providing limited transparency regarding the rationale behind diagnostic decisions, a challenge also highlighted in [45]. Some XAI techniques have attempted to improve transparency—for example, decoding the inner layers of the neural network using principal component analysis and model-specific interpretation strategies [46]—however, these approaches still struggle to produce clinically actionable interpretations. Previous research has also shown that techniques not tied to a specific model can predict outcomes [23], but may fail to align important regions with known biomarkers, reducing clinical confidence.

Furthermore, many studies have focused on whole MRI sequences, which has increased the opacity of the model and the computational load without improving interpretability. The ongoing tension between achieving high accuracy and maintaining explainable interpretations—as identified in [14] further limits the reliability of current methods, especially for general practitioners or non-specialists who require intuitive reasoning and a clinical basis.

As a result, current XAI approaches fail to provide integrated, reliable, and clinically meaningful interpretations consistent with known biomarkers of AD. This study aims to solve this problem by developing an interpretable hybrid framework that produces quantitative clinical interpretations and spatial evidence from MRI within a unified diagnostic pathway.

## 4. Research Objective

The primary objective of this study is to develop and evaluate an HXAI framework for the diagnosis of AD through the integration of both model-agnostic and model-specific. Unlike existing approaches that rely solely on either clinical data or MRI scans, this study integrates both modalities to improve diagnostic explainability accuracy and interpretability. The framework specifically aims to implement a dual-layer explainability approach by combining rule-based explanations (SHAP, a model-agnostic technique) for clinical data with visual explanations (Grad-CAM, a model-specific technique) for MRI, addressing the limitations of prior studies that used only one explanation method or lacked clinical grounding. This study validates the explainability accuracy through quantitative methods, including SHAP removal-based analysis and Grad-CAM IoU. The framework seeks to bridge the gap between accuracy and interpretability, ultimately improving explainability accuracy and providing a trustworthy AI tool that enhances physician confidence and supports practical clinical adoption in early AD diagnosis.

## 5. Hybrid Explainable AI Framework (HXAI)

**Figure 1:**
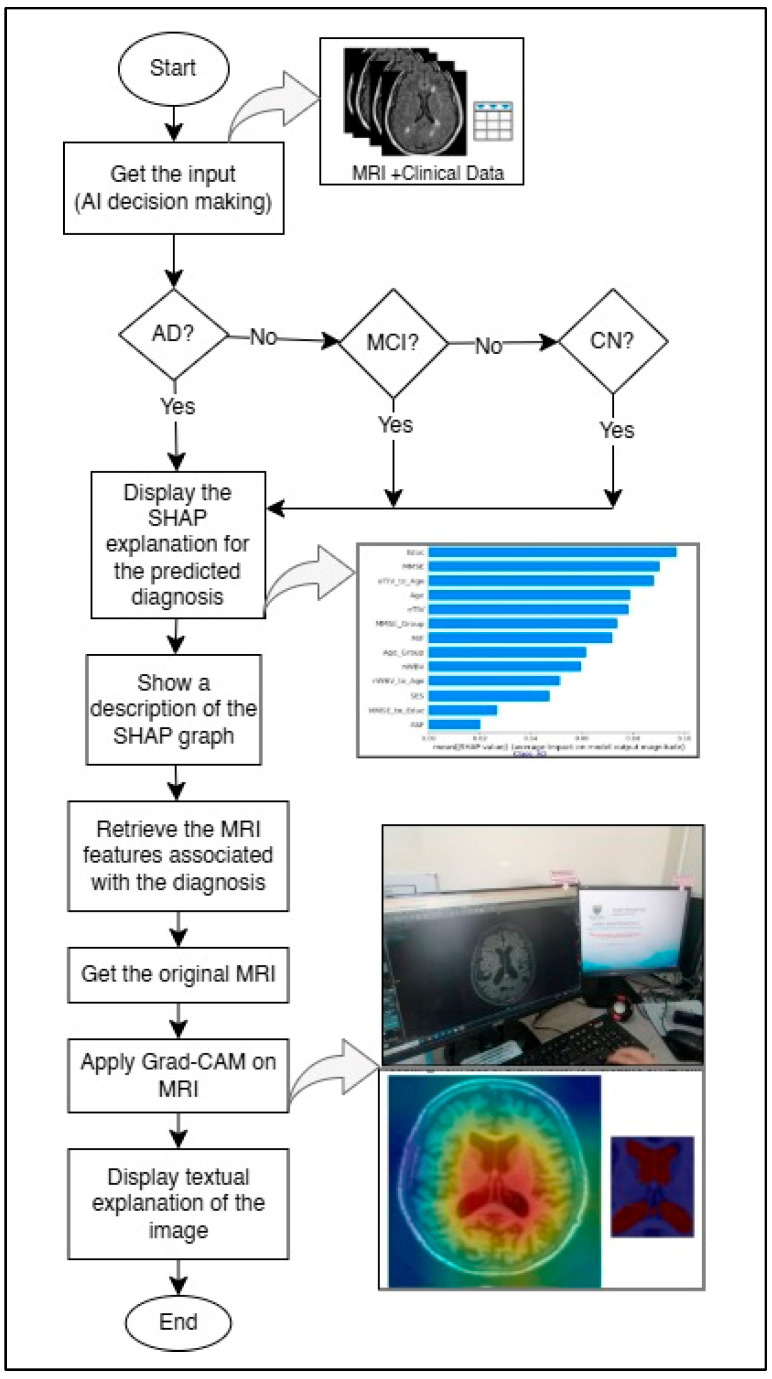
Proposed HXAI workflow combining SHAP explanations and Grad-CAM visualizations for AD diagnosis.

This section details the method used to enhance explainability, referred to as “HXAI”. After training the model to detect AD using the middle slices showing the lateral ventricles and ensuring high accuracy using a metamodel [12], the next step is to explain the decision-making process, as shown in Figure 1. To achieve this, (1) the decision result from the pre-trained model is displayed with the percentage of confidence. (2) The clinical data for the predicted classification, which matches the input clinical data, is then displayed using SHAP (a model-agnostic technique), and the rationale is explained. Because the model was trained on the middle slices [12,47] that clearly show the lateral ventricles, (3) the lateral ventricles are highlighted in heatmaps using Grad-CAM (a model-specific technique) to improve explainability, and the rationale for the classification based on the size of the lateral ventricles is explained.

### 5.1. Clinical-Based Feature Attribution

The SHAP-based feature attribution was applied to quantify the contribution of each clinical feature to the classification decisions. The inputs for this module included 14 primary clinical features (e.g., Mini-Mental State Examination (MMSE), normalized Whole Brain Volume (nWBV), Clinical Dementia Rating (CDR), age, estimated Total Intracranial Volume (eTIV), and Atlas Scaling Factor (ASF)), along with several derived features representing ratios or groupings (e.g., eTIV_to_Age, MMSE_to_Educ).

SHAP values were computed for all samples in the test set using the model’s predicted probability. For each class (CN, MCI, AD), the absolute SHAP values were calculated across all samples to identify the most influential features for that class. Summary plots and bar charts were generated to visualize the impact of each feature on the model’s output.

Inputs: Clinical features from the dataset *(X_combined)*.

Outputs: SHAP values per feature for each class; visualizations including summary plots, class-specific feature importance, and top 10 feature rankings.

The process began by loading the trained model and preparing the test dataset, which included all relevant clinical variables such as MMSE, nWBV, Age, Gender, and EDUC. The input features were first normalized using *StandardScaler()* to ensure that all variables were within the same numerical range. Following this, a SHAP explainer object was created using the trained model through the command *shap.Explainer(model, data)*, which automatically selects the most suitable explanation algorithm depending on the model type.

Next, SHAP values were computed for each observation in the test set using the method .*shap_values(X_test)*. These SHAP values represented the *marginal contribution* of each clinical feature to the model’s output for each patient. A global summary plot was then generated using *.summary_plot()* to display the overall importance of features across all test samples, while individual force plots *(.force_plot())* were used to show case-specific explanations.

SHAP identified that MMSE score and normalized whole-brain volume (nWBV) had the highest positive contributions toward distinguishing between CN, MCI, and AD. Age showed a moderate but consistent influence, while demographic factors such as Gender and EDUC contributed marginally. At the local level, the SHAP visualization enabled the model to highlight, for each case, whether an increased or decreased value in a specific feature pushed the prediction toward AD or CN.

The textual explanation was automatically generated alongside the plots to explain each feature’s role. For example, lower MMSE values or reduced nWBV were described as increasing the probability of AD classification. This multi-level interpretation enabled clinicians to understand not only which features influenced the decision but also how and why these features shaped the model’s reasoning.

### 5.2. Neuroimaging-Based Feature Segmentation

Grad-CAM was applied to the MRI-based model to provide a visual explanation of the regions in the brain that contributed most to the classification. The feature extractor was based on a pre-trained ResNet50 model, and the last convolutional layer *(conv5_block3_out)* was selected to compute the Grad-CAM heatmaps.

For each MRI image, the gradient of the predicted class with respect to the feature maps of this last convolutional layer was calculated. These gradients were pooled and combined with activation maps to generate a class-discriminative heatmap. The heatmap was then overlaid on the original MRI image to highlight critical regions.

Inputs: Preprocessed MRI images from the test set.

Outputs: Grad-CAM heatmaps, overlaid images showing the most influential regions, and highlighted lateral ventricle areas.

The process started with the trained CNN model (ResNet50) used for feature extraction from the middle MRI slices. Using the *TensorFlow GradientTape()* environment, gradients were calculated with respect to the final convolutional layer. Specifically, during backpropagation, the gradients of the class score (for CN, MCI, or AD) were computed with respect to each feature map activation in the target convolutional layer. These gradients were then pooled using the *tf.reduce_mean()* operation to obtain the importance weights for each channel in the feature map.

The weighted combination of these activations and the pooled gradients was computed to generate a *coarse localization map* that highlights the region’s most influential in the classification process. This map was then resized to the original MRI dimensions using OpenCV’s *cv2.resize()* and normalized between 0 and 1. Finally, the heatmap was superimposed onto the original MRI slice using *matplotlib’s colormap overlay* to visually emphasize high-activation regions associated with AD progression.

Since the model was trained exclusively on middle MRI slices showing the lateral ventricles, the resulting Grad-CAM maps consistently highlighted these areas as primary regions contributing to the AD classification.

This visual interpretability allowed medical users to verify that the model’s attention was focused on clinically valid anatomical regions. The inclusion of these Grad-CAM heatmaps in the interface provided both transparency and trustworthiness, enabling non-expert clinicians to visually understand why a certain decision (e.g., AD, MCI, or CN) was made. Moreover, the textual summary accompanying each heatmap described the anatomical relevance of the highlighted region, reinforcing interpretability at both the visual and conceptual levels. Overall, the Grad-CAM module transformed abstract CNN activations into human-understandable explanations, strengthening clinical confidence in model reliability and ensuring alignment between the model’s reasoning and medical knowledge.

### 5.3. Clinical and Neuroimaging Integration in HXAI

To address the unclear nature of current XAI, this study combines the SHAP and Grad-CAM models to improve explainability. While Grad-CAM identifies the most influential regions in MRI images (on which the model was primarily trained), SHAP analyzes the clinical features that contribute to classification. In this study, we term this approach “HXAI”, combining visual explanations with clinical calculations and written explanations to support decision-making. It is important to clarify that the integration between SHAP and Grad-CAM in HXAI occurs only at the presentation level, where both explanation outputs are displayed together to provide a unified interpretive view.

Identify only the middle slices of the MRI: The pre-trained AI model focuses on analyzing the middle slices of the MRI that show the lateral ventricles, reducing the noise generated by using all slices. The model should be made to focus on only one level of importance in detecting AD to support subsequent decision-making.

Identifying key clinical features: Clinical features that are clinically essential in detecting AD are identified.

Integrating visual explanations with relevant information: The model’s explanation highlights the identified regions of interest and supports the explanation with textual information explaining the identified region in the MRI and clinical data to support decision-making.

Improving decision support: This approach allows clinicians and non-clinicians to correlate visual evidence with clinical measurements, enhancing confidence in the decisions made by the model.

Unlike existing XAI that focuses only on presenting AI decisions and identifying areas outside the brain as important in the decision-making process without supporting them with additional information, HXAI provides a deeper level of transparency and clarity, and facilitates the use of models in clinical settings.

## 6. Data Sources and Preprocessing

The primary dataset used in this study is the Alzheimer’s Disease Neuroimaging Initiative (ADNI), one of the most well-known and established resources in AD research. ADNI provides high-quality structural MRI scans, along with comprehensive clinical information, making it particularly suitable for reproducible and comparable studies. The dataset covers a wide spectrum of patient demographics and disease stages, ensuring that the results are clinically relevant and generalized. The dataset used in this study consists of 1568 subjects, including CN, MCI, and AD cases. It contains 1.5 T and 3 T T1-weighted MRI scans, along with comprehensive clinical assessments that include the MMSE, CDR, neuropsychological test batteries, age, sex, and educational level.

To ensure data quality, unusable or corrupted images were excluded. The data and preprocessing steps were verified with a geriatrician, Associate Prof. Dr. Khor Hui Min. Preprocessing involved several steps: (1) normalizing MRI pixel values to a standard intensity range; (2) resizing images to a standard dimension of 224 × 224 pixels, consistent with the input requirements of ResNet50; and (3) data augmentation techniques, including rotation, flipping, and zooming, to increase the diversity of datasets and reduce overfitting.

We first used the full MRI images and split the data into 80% for training and 20% for testing, and the reason for using the full MRI was to verify the performance of the models used. After that, we selected the mid-slice and combined it with the clinical data, and we applied k-fold cross-validation using five folds. The reason for using k-fold was to provide a more reliable and stable evaluation due to the smaller size of the combined dataset and to ensure that all samples are used for both training and validation.

## 7. Experiment Setup

In this section, we analyze and evaluate the effectiveness of the explanations provided by our framework. These explanations include clinical feature attributions generated using a model-agnostic (SHAP) and visual heatmaps generated using a model-specific (Grad-CAM). This comparison will be supported by published studies and specialized research in the field of XAI to determine how clear and easy to understand the explanations provided by our model are, and how useful they are in enhancing physician confidence.

### 7.1. Feature Extraction and Base Models

Clinical features, including MMSE, age, gender, and nWBV, were represented as structured numerical data. Mid-slices from MRI scans showing the lateral ventricles were selected, normalized, resized, and augmented to enhance dataset diversity. Imaging features were extracted using a ResNet-50 model pre-trained on ImageNet, with the final fully connected layers removed to generate 2048-dimensional feature vectors that capture spatial and structural information.

Four base classifiers (RF, XGB, SVM, GB) were trained independently on the extracted features, and their predictions were combined using a logistic regression meta-model. Class weights were applied to address imbalance across AD, MCI, and CN categories. To ensure evaluation consistency across all models, predefined parameter settings for the four base classifiers were used instead of performing hyperparameter optimization. These settings were chosen based on values commonly recommended from previous studies and preliminary experiments to verify model behavior rather than maximizing performance. The meta-model (logistic regression) was used with default parameters. No hyperparameter optimization was applied, as the main objective at this stage was to evaluate the comparative performance and stability of the models under standardized and controlled settings. This stacked machine learning framework, described in greater detail in our previous work [12], forms the predictive core of the proposed HXAI approach by integrating multimodal features while maintaining high diagnostic accuracy and robustness. Having established predictive architecture, the next step focuses on explainability.

### 7.2. Clinical Data Analysis

To enhance the explainability of the clinical component of the model, SHAP was applied to quantify the relative importance of individual features in predicting AD, MCI, and CN. This approach provides a transparent mapping between input variables and the model’s output, thereby addressing the clinical need for understanding why a particular diagnosis was made.

#### 7.2.1. Mean Degree of Change Measures (MDMC)

According to [48], the MDMC is a methodology used to evaluate the effectiveness of XAI techniques. To apply MDMC to SHAP, we modify the test data based on the importance of the features previously identified by SHAP and then re-evaluate the model’s performance after the modification. This method aims to test how well XAI interprets the model’s decisions. The test data is perturbed by removing or modifying the values of previously identified important features. We then re-evaluate the model after the data modification and compare the new performance to the original performance.

#### 7.2.2. Feature Importance Analysis

Visualization techniques provide insights into how the model makes decisions by highlighting the most influential features in the image. This makes it easier to verify whether the model is relying on clinically relevant information. The explainability of the model’s decision-making process can be assessed by analyzing these explanations and ensuring they align with clinical understanding in AD diagnosis.

**Figure 2:**
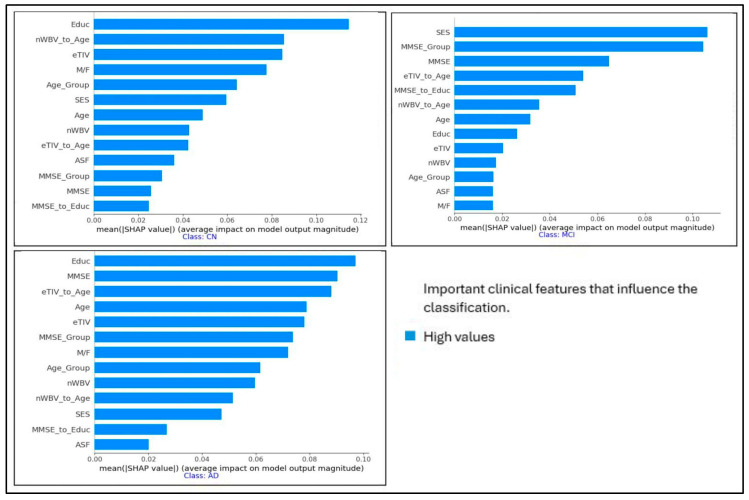
SHAP summary plots for the most important clinical features that influence the classification of individuals with AD, MCI, and CN.

The SHAP summary plots in Figure 2 show the most important clinical features that impact the classification of individuals with AD, mild cognitive impairment (MCI), and cognitively normal (CN). Each plot corresponds to one of the three categories and shows the impact of individual features on the model’s performance. The *x*-axis represents the SHAP values, indicating whether the feature drives the prediction toward or away from a particular category. In the AD category, Educ, MMSE, and eTIV_to_Age are among the most influential features, while SES, MMSE, and eTIV_to_Age play a critical role in the MCI category. The CN classification emphasizes Educ, nWBV_to_Age, and eTIV. These insights help in understanding the contribution of different features to the decision-making process of the model, enhancing explainability in the diagnosis of AD. We use removal-based explanations to measure the explainability of our results. Removal-based explanations are particularly useful for validating explanation methods, as they provide empirical evidence about whether specific important features influence predictions.

#### 7.2.3. Clinical-Based Explanation

Removal-based explanations are a core approach in XAI, assessing the significance of features by totally removing them and assessing their impact on model performance. The explainability of SHAP values was further evaluated using a removal-based verification process, in which features identified as most important were systematically excluded and the model’s performance was re-assessed. This approach builds on the general framework of removal-based explanations introduced by [49], where the predictive value of a subset of features S is formalized as:

(4) $$v\left(S\right)=E\left[f\left(x\right)|x_{S}\right]$$

Here, f(x) denotes the model, x_S represents the input restricted to the subset of features S , and v(S) captures the expected output of the model given only those features. To adapt this framework into practice, we measured the change in classification accuracy after removing the most influential features, as determined by SHAP values:

(5) $$\Delta Acc=Acc_{original}-Acc_{removal}$$

where $Acc_{original}$ is the model accuracy before feature removal, and $Acc_{removal}$ is the accuracy after removing the top-ranked features.

### 7.3. Neuroimaging Visualization Analysis

To interpret the imaging component of the model, Grad-CAM was employed to generate visual heatmaps highlighting regions of interest in the mid-slice MRI scans. This technique provides spatial explanations by indicating which areas of the brain most strongly influenced the model’s predictions.

The researchers [41,50] found that Grad-CAM is reliable at identifying important parts of images, but it is not always perfect. Grad-CAM outperforms CAM because it works with any network, not just those with global average pooling layers. However, it can be sensitive to some changes in the input and sometimes highlights imperfect regions. Therefore, a measure of localization accuracy is used to evaluate how well Grad-CAM can identify the correct regions. This requires calculating the Intersection over Union (IoU) between the Grad-CAM map and the true bounding box of the target in the image. Following the common practice in published studies [50], an IoU threshold of 50% was considered the minimum acceptable overlap between the Grad-CAM map and the ground truth bounding box to qualify as a successful explanation.

A Bounding Box is a rectangle that surrounds a target object within an image. This rectangle is used to locate the object and is often represented by the following coordinates:

(6) $$\left(x_{\left\{min\right\}},y_{\left\{min\right\}},x_{\left\{max\right\}},y_{\left\{max\right\}}\right)$$

where $x_{\left\{min\right\}},y_{\left\{min\right\}}$ represents the upper-left corner of the box, and $x_{\left\{max\right\}},y_{\left\{max\right\}}$ represents the lower-right corner. The more overlap between the Grad-CAM map and the bounding box, the more accurate the explanations.

**Figure 3:**
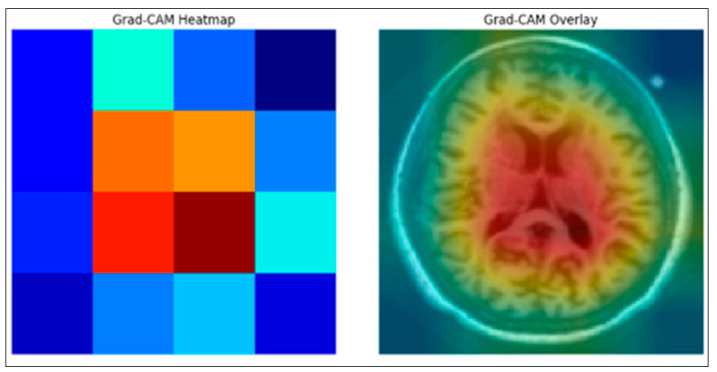
Grad-CAM Ground truth Overlap of the proposed Enhanced XAI.

The mid-slice MRI image was first passed through the trained ResNet50 model to obtain feature maps from the final convolutional layer. The Grad-CAM weights were then calculated by applying global mean aggregation to the gradients associated with the predicted class. These weights were multiplied by the corresponding feature maps to produce the Grad-CAM primary heat map. The heat map was normalized and visualized as a color-coded activation map, as shown in the left image of Figure 3. For the synthesis, the Grad-CAM map was resized to match the resolution of the original MRI and combined with the input mid-slice MRI image to highlight the brain regions that contributed most to the model’s prediction, as shown in the right-hand image of Figure 3. We calculated the explanation accuracy by using the Grad-CAM heatmap of the image and defined the true region of interest (Ground Truth Bounding Box). We then calculated the overlap between Grad-CAM and the bounding box using IoU. Calculating the proportion of images with an IoU above a certain threshold is a measure of explainability.

## 8. Real-Time Performance Evaluation Results

The proposed framework, which integrates clinical feature attribution and neuroimaging visualizations in a single framework, provides a comprehensive and interpretable AI system for AD diagnosis. In our earlier study [12], the underlying framework achieved high classification accuracy on MRI data (ADNI = 98.62%). To evaluate the reliability of the feature explanations, the MDMC test (as mentioned in Section 7.2.1) was applied. MDMC checks if the features found by SHAP are truly important by removing them and observing the effect on model accuracy. With all features present, the model achieved 98.62%. After MDMC removed the top features highlighted by SHAP as most important, the model’s accuracy dropped significantly to 43.98%. This demonstrates that the explanations provided by SHAP are not random; they correctly identify the clinical features that the model relies on for accurate diagnosis. Furthermore, the Feature Importance Analysis provided valuable insights into how the model makes decisions by highlighting the most influential features, facilitating verification that the model relies on clinically relevant information.

Additionally, MRI-based visualization analysis using Grad-CAM (a model-specific method) demonstrated an explanation accuracy of 58.16% when compared to clinically relevant regions, confirming that the model’s highlighted areas reasonably match actual disease-affected regions. Together, these results demonstrate that the proposed framework not only enables the model to achieve high accuracy but also provides multi-level interpretability, feature-level via SHAP and image-level via Grad-CAM, enhancing transparency, explainability, and clinical trust in AD diagnosis.

**Figure 4:**
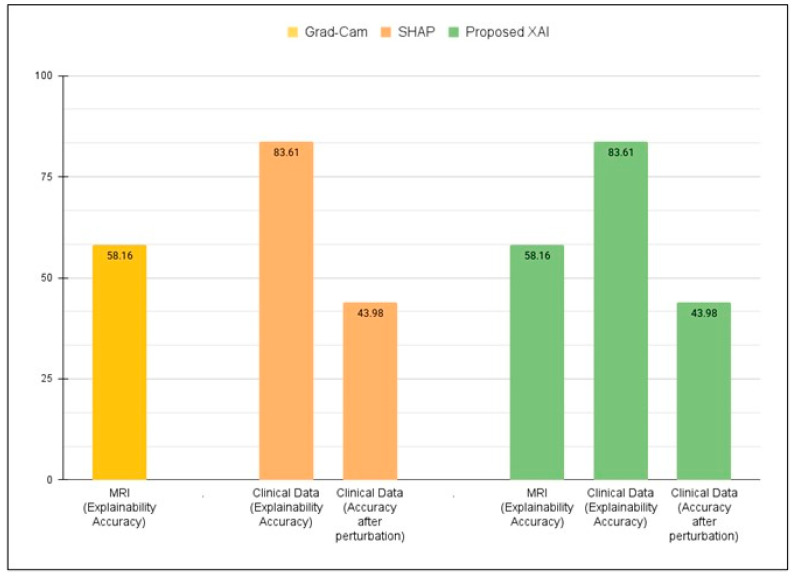
Quantitative validation of explainability using SHAP (clinical data), Grad-CAM (MRI), and the proposed dual-layer XAI approach.

Figure 4 illustrates the validity of interpretability across three settings. Grad-CAM applied to MRI alone achieved 58.16%, while SHAP applied to clinical data achieved 83.61%, dropping to 43.98% after feature removal. The proposed XAI approach combined both methods, maintaining the same interpretability accuracy but improving consistency and clinical explainability compared to single-modality methods.

**Figure 5:**
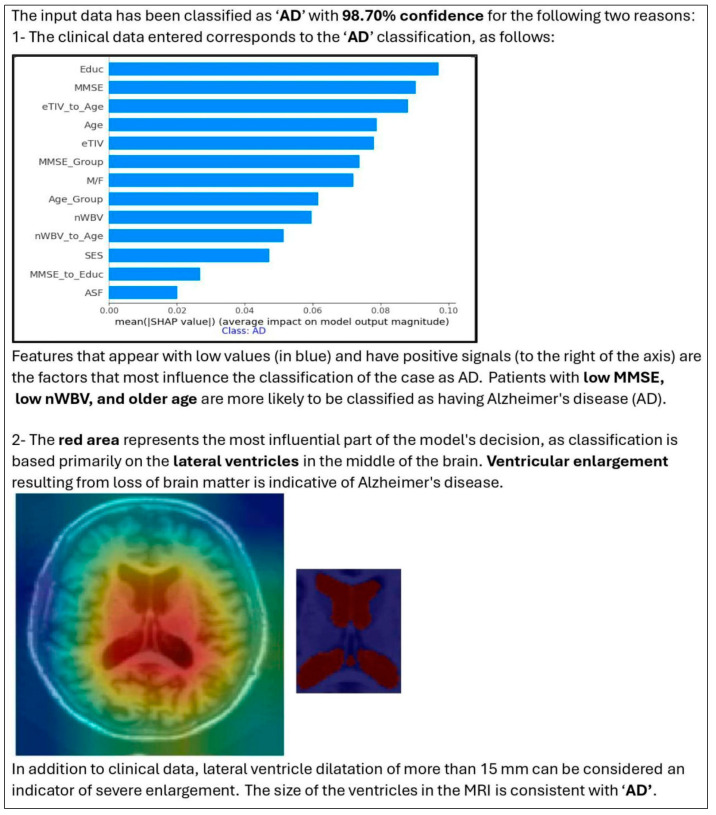
Outputs of the proposed HXAI framework, integrating SHAP-based clinical explanations and Grad-CAM MRI visualizations within a stacked ensemble learning model.

The combination of SHAP (for the importance of clinical features) and Grad-CAM (for highlighting MRI regions) provided a more comprehensive explanation of model decisions. The HXAI approach, combining visual and quantitative annotation, improves transparency and clinical relevance, addressing the “black box” limitations of traditional AI models. Figure 5 shows an example of how the visualization of the proposed Enhanced XAI helps in model evaluation. The figure shows an MRI slice of a patient that the model has rated, and the model then shows the decision confidence along with why the AI made that decision.

The image on the far left shows the original MRI scan, while the middle image overlays a highlighted region corresponding to the volume of the ventricle that is automatically segmented. This visualization confirms that the model considers clinically relevant brain structures in its classification process. By incorporating such visual explanations, our model ensures that its decisions are consistent with clinical reasoning. The HXAI framework provides clinicians with both visual evidence (Grad-CAM heatmaps) and quantitative feature contributions (SHAP), facilitating more informed decisions.

## 9. Discussions

While studies using a model-agnostic method or a model-specific method provided partial explanations, they either lacked quantitative validation or clinical grounding. In contrast, our framework focused on clinically meaningful mid-slices, improving both interpretability and computational efficiency compared to prior works that trained on full MRI sequences.

Our proposed HXAI framework demonstrates significant improvements over traditional methods in terms of clinical relevance. Specifically:

1. Selecting clinically meaningful MRI slices reduces complexity, improves computational efficiency, and maintains clinical relevance.
2. Combining clinical features with MRI-derived characteristics in a single framework leads to superior diagnostic confidence in Alzheimer’s disease compared to using either modality alone, thereby adding credibility to the internal reasoning of the model.
3. Using both SHAP (for clinical data) and Grad-CAM (for MRI) provides multi-layered explanations, helping physicians understand what the model predicts and why.
4. By aligning visual explanations (Grad-CAM) and feature-based explanations (SHAP) with established medical knowledge, the framework provides transparent reasoning, addressing one of the main barriers to clinical adoption of AI.
5. SHAP removal-based analysis and Grad-CAM IoU metrics show that the produced explanations are not random but correspond to relevant features and important brain regions.
6. The framework is designed to assist general practitioners and non-neurologists in understanding diagnostic decisions, which may increase the adoption of AI in the absence of specialists and in resource-limited settings.
7. Given its efficiency and interpretability, the HXAI model can be integrated into MRI machines, providing instant and interpretable diagnostics during the scanning process.
8. The architecture of the framework indicates the possibility of deployment on mobile devices or in low-resource environments, expanding access to explainable AI beyond specialized clinical settings.

In summary, the findings demonstrate that the dual-layer approach in the HXAI framework, which has been quantitatively validated through SHAP removal-based tests and Grad-CAM IoU analysis, directly addresses the limitations of previous XAI methods [51], which often fail to provide actionable clinical insights. Consequently, the framework ultimately improves explainability accuracy in an accurate and interpretable way and strengthens trust in AI-assisted diagnosis.

While the HXAI framework demonstrates high accuracy in diagnosing AD, it is important to note that the current study specifically focuses on differentiating AD from MCI and AD stages within the AD spectrum. The framework has not yet proven effective against other dementia-related diseases or other conditions that may be accompanied by similar structural changes, such as frontotemporal dementia or Lewy body dementia. This represents a limitation on the current diagnostic accuracy of our model. Future research will focus on expanding the framework’s differential diagnostic capabilities by incorporating additional biomarkers and training on multi-category datasets encompassing a broader spectrum of neurodegenerative diseases.

## 10. Conclusions

This study introduced an HXAI framework that integrates model-agnostic clinical explanations (via SHAP) and model-specific visual explanations (via Grad-CAM). By combining rule-based explainability with example-based visualizations, the framework enhances transparency and trustworthiness in AI-assisted diagnosis of AD.

The underlying model is responsible for making the actual AD predictions, while the framework provides a structure that combines the model’s predictions with interpretable tools, enabling clinicians to obtain both quantitative assessments of feature importance and visual confirmations that the model focuses on medically relevant brain regions, such as the lateral ventricles. Key contributions of this study include the following:

1. A multimodal framework that integrates clinical features and MRI mid-slices, capturing structural ventricular changes critical to AD.
2. Employing ensemble learning with a meta-model to improve the model’s robustness and diagnostic accuracy.
3. Demonstrating that the proposed XAI framework improves predictive performance and enhances explainability accuracy, addressing the limitations of existing XAI methods by offering validated, clinically meaningful explanations.

Through quantitative validation, the HXAI approach has proven effective in bridging the gap between model accuracy and interpretability, ultimately achieving higher explainability accuracy and positioning itself as a practical and trustworthy tool for early AD diagnosis.

### Future Work

Despite promising results, some limitations remain. Future work will focus on extending validation to additional datasets and multi-center studies to ensure broader applicability. Although our framework, trained solely on the middle slices, demonstrated high accuracy in diagnosing AD, from a clinical perspective, dilated lateral ventricles may be indicative of problems other than AD. In the future, multiple models could be trained, each focusing on a single region of interest for AD diagnosis on MRIs. The individual models can be integrated into a single framework that generates multiple explanations. Each explanation reflects the region analyzed by its respective model, while accompanying textual information is provided to support clinical decision making.

The current HXAI framework relies on structural changes in MRI and clinical assessments rather than direct measurement of amyloid beta or tau levels. While the ventricular hypertrophy captured by our MRI analysis and the cognitive decline reflected in clinical features such as MMSE are well-known indicators of AD progression, they represent aftereffects of the underlying disease. Future versions of this framework can be improved by incorporating positron emission tomography (PET) data or cerebrospinal fluid (CSF) biomarkers to provide a more comprehensive pathological correlation and enable early detection through direct assessment of amyloid-beta and tau disease.

The HXAI framework can also be extended to differentiate between early-onset and late-onset AD. These subtypes exhibit different patterns of structural changes in the brain and clinical symptoms, particularly with respect to age. Because the current model incorporates both MRI-derived features and clinical features such as MMSE and age, it has the potential to support this distinction once age-disaggregated datasets are available (e.g., under 65 years vs. ≥65 years). Integrating this data would enable the framework to provide more accurate and personalized diagnostic insights.

## Figures and Tables

**Table 1:** Analysis of Explanation Methods and Performance Trade-offs in Previous AD Detection Studies.

Author	Diagnostic Performance	Primary XAI Focus	Key Limitation
[33]	High 98%	Model-specific	Uses a simple, explainable model that likely cannot capture the complex patterns in fMRI data.
[26]	High 88.4%	Model-specific	Rely only on visual explanations without text-based or quantitative reasoning, limiting its utility for comprehensive clinical reporting.
[37]	High 98.6%	Model-agnostic	SHAP provides feature significance but fails to visually identify affected brain regions on MRI scans, making interpretation difficult to interpret intuitively.
[17]	High 96.8%	Model-agnostic	The explanations provided are insufficient to comprehensively understand the complex decision-making process of the multimodal model.
[38]	High 92.56%	Model-specific	Provides no specific XAI technique; the model remains a black box with no insight into which facial features drive the decision.
[16]	High 89%	Model-agnostic	Cannot generate visual heatmaps to pinpoint the exact anatomical regions in the MRI scans.
[36]	Moderate 71.4%	Model-agnostic	Focuses on neuron-level details without linking them to a global, model-agnostic feature importance that is more easily understood by clinicians.
[19]	High 99.9%	Model-agnostic	Cannot generate visual heatmaps to pinpoint the exact anatomical regions in the MRI scans.
[35]	High 89.02%	Model-agnostic	Provides visual explanations but lacks complementary, model-agnostic reasoning based on clinical data.
[24]	High 86.82%	Model-agnostic	Explanations are approximate and unstable, as they do not accurately represent the complex decision-making process of the CNN model on MRI data.
Our work	Target: High F1-Score	Integrated (Model-Agnostic and Model-Specific)	Aims to resolve these trade-offs by combining SHAP (for clinical data) and Grad-CAM (for MRI) in a unified, trustworthy interface.

## Data Availability

The data used in this study are publicly available. Data from the Alzheimer’s Disease Neuroimaging Initiative (ADNI) are accessible upon registration and approval via the ADNI website at http://adni.loni.usc.edu (accessed on 8 June 2024). The Open Access Series of Imaging Studies (OASIS) datasets are freely available through https://www.oasis-brains.org/ (accessed on 21 January 2025). The source code used to preprocess the data, train the models, and generate the diagnostic explanations is publicly available at the following GitHub repository: https://github.com/F-H5/diagnosis_explanation_module (accessed on 15 May 2025).

## Abbreviations

The following abbreviations are used in this manuscript:

AD	Alzheimer’s Disease
ADNI	Alzheimer’s Disease Neuroimaging Initiative
CNN	Convolutional Neural Network
CN	Cognitively Normal
DT	Decision Tree
GB	Gradient Boosting
Grad-CAM	Gradient-weighted Class Activation Mapping
HXAI	Hybrid Explainable AI
IoU	Intersection over Union
LIME	Local Interpretable Model-Agnostic Explanations
LR	Logistic Regression
LRP	Layer-wise Relevance Propagation
MCI	Mild Cognitive Impairment
MDMC	Mean Degree of Change Measures
MMSE	Mini-Mental State Examination
MRI	Magnetic Resonance Imaging
OASIS	Open Access Series of Imaging Studies
PDA	Pixel Density Analysis
PGM	Probabilistic Graphical Model
RF	Random Forest
SHAP	SHapley Additive exPlanations
SMOTE	Synthetic Minority Over-sampling Technique
SVM	Support Vector Machine
XAI	Explainable Artificial Intelligence
XGB	Extreme Gradient Boosting

## References

[1] Arya A.D., Verma S.S., Chakarabarti P., Chakrabarti T., Elngar A.A., Kamali A.-M., Nami M. (2023). A systematic review on machine learning and deep learning techniques in the effective diagnosis of Alzheimer’s disease. Brain Inform..

[2] Peter O.E.A., Chellappa R., Choudhry N., Demiris G., Ganesan D., Karlawish J., Marlin B., Li R.M., Dehak N., Arbaje A. (2024). Artificial Intelligence and Technology Collaboratories: Innovating aging research and Alzheimer’s care. Alzheimer’s Dement..

[3] Villain N., Fouquet M., Baron J.-C., Mézenge F., Landeau B., de La Sayette V., Viader F., Eustache F., Desgranges B., Chételat G. (2010). Sequential relationships between grey matter and white matter atrophy and brain metabolic abnormalities in early Alzheimer’s disease. Brain.

[4] Chi-Ping T.M.-C.M., Chang H.-I., Huang C.-W., Chou M.-C., Chang C.-C. (2024). Diet Pattern Analysis in Alzheimer’s Disease Implicates Gender Differences in Folate–B12–Homocysteine Axis on Cognitive Outcomes. Nutrients.

[5] Zakaria M.M.A., Doheir M., Akmaliah N., Yaacob N.B.M. (2024). Infinite potential of ai chatbots: Enhancing user experiences and driving business transformation in e-commerce: Case of Palestinian e-commerce. J. Ecohumanism.

[6] Vrahatis A.G., Skolariki K., Krokidis M.G., Lazaros K., Exarchos T.P., Vlamos P. (2023). Revolutionizing the Early Detection of Alzheimer’s Disease through Non-Invasive Biomarkers: The Role of Artificial Intelligence and Deep Learning. Sensors.

[7] Khan P., Kader F., Islam S.M.R., Rahman A.B., Kamal S., Toha M.U., Kwak K.-S. (2021). Machine Learning and Deep Learning Approaches for Brain Disease Diagnosis: Principles and Recent Advances. IEEE Access.

[8] Ritchie C.W., De la Fuente Garcia S., Luz S. (2020). Artificial Intelligence, Speech, and Language Processing Approaches to Monitoring Alzheimer’s Disease: A Systematic Review—PubMed. J. Alzheimer’s Dis. JAD.

[9] Neji M., Hassen S.B., Hussain Z., Hussain A., Alimi A.M., Frikha M. (2024). Deep learning methods for early detection of Alzheimer’s disease using structural MR images: A survey. Neurocomputing.

[10] Razaque A., Bazarbekov I., Ipalakova M., Yoo J., Assipova Z., Almisreb A. (2024). A review of artificial intelligence methods for Alzheimer’s disease diagnosis: Insights from neuroimaging to sensor data analysis. Biomed. Signal Process. Control.

[11] Fujita K., Katsuki M., Takasu A., Kitajima A., Shimazu T., Maruki Y. (2022). Development of an artificial intelligence-based diagnostic model for Alzheimer’s disease. Aging Med..

[12] Al-Bakri F.H., Bejuri W.M.Y.W., Al-Andoli M.N., Ikram R.R.R., Khor H.M., Tahir Z., The Alzheimer’s Disease Neuroimaging Initiative (2025). A Meta-Learning-Based Ensemble Model for Explainable Alzheimer’s Disease Diagnosis. Diagnostics.

[13] Angelov P.P., Soares E.A., Jiang R., Arnold N.I., Atkinson P.M. (2021). Explainable artificial intelligence: An analytical review. Wiley Interdiscip. Rev. Data Min. Knowl. Discov..

[14] Viswan V., Shaffi N., Mahmud M., Subramanian K., Hajamohideen F. (2023). Explainable Artificial Intelligence in Alzheimer’s Disease Classification: A Systematic Review. Cogn. Comput..

[15] Kamal M.S., Chowdhury L., Nimmy S.F., Rafi T.H.H., Chae D.K. An Interpretable Framework for Identifying Cerebral Microbleeds and Alzheimer’s Disease Severity using Multimodal Data. Proceedings of the 2023 45th Annual International Conference of the IEEE Engineering in Medicine & Biology Society (EMBC).

[16] Haddada K., Khedher M.I., Jemai O., Khedher S.I., El-Yacoubi M.A. Assessing the Interpretability of Machine Learning Models in Early Detection of Alzheimer’s Disease. Proceedings of the 2024 16th International Conference on Human System Interaction (HSI).

[17] Jahan S., Abu Taher K., Kaiser M.S., Mahmud M., Rahman S., Hosen A.S.M.S., Ra I.-H. (2023). Explainable AI-based Alzheimer’s prediction and management using multimodal data. PLoS ONE.

[18] Xu X., Yan X. A Convenient and Reliable Multi-Class Classification Model based on Explainable Artificial Intelligence for Alzheimer’s Disease. Proceedings of the 2022 IEEE International Conference on Advances in Electrical Engineering and Computer Applications (AEECA).

[19] Yilmaz D. Development and Evaluation of an Explainable Diagnostic AI for Alzheimer’s Disease. Proceedings of the 2023 International Conference on Artificial Intelligence Science and Applications in Industry and Society (CAISAIS).

[20] Salvatore C., Battista P., Berlingeri M., Cerasa A., Castiglioni I. (2020). Artificial intelligence and neuropsychological measures: The case of Alzheimer’s disease. Neurosci. Biobehav. Rev..

[21] Al Olaimat M., Martinez J., Saeed F., Bozdag S., Initiative A.D.N. (2023). PPAD: A deep learning architecture to predict progression of Alzheimer’s disease. Bioinformatics.

[22] Brusini L., Cruciani F., Dall’aGlio G., Zajac T., Galazzo I.B., Zucchelli M., Menegaz G. (2024). XAI-Based Assessment of the AMURA Model for Detecting Amyloid-β and Tau Microstructural Signatures in Alzheimer’s Disease. IEEE J. Transl. Eng. Health Med..

[23] Deshmukh A., Kallivalappil N., D’SOuza K., Kadam C. AL-XAI-MERS: Unveiling Alzheimer’s Mysteries with Explainable AI. Proceedings of the 2024 Second International Conference on Emerging Trends in Information Technology and Engineering (ICETITE).

[24] Shad H.A., Rahman Q.A., Asad N.B., Bakshi A.Z., Mursalin S., Reza T., Parvez M.Z. Exploring Alzheimer’s Disease Prediction with XAI in various Neural Network Models. Proceedings of the TENCON 2021–2021 IEEE Region 10 Conference (TENCON).

[25] Vimbi V., Shaffi N., Mahmud M. (2024). Interpreting artificial intelligence models: A systematic review on the application of LIME and SHAP in Alzheimer’s disease detection. Brain Inform..

[26] Tima J., Wiratkasem C., Chairuean W., Padongkit P., Pangkhiao K., Pikulkaew K. Early Detection of Alzheimer’s Disease: A Deep Learning Approach for Accurate Diagnosis. Proceedings of the 2024 21st International Joint Conference on Computer Science and Software Engineering (JCSSE).

[27] Mansouri D., Echtioui A., Khemakhem R., Hamida A.B. Explainable AI Framework for Alzheimer’s Diagnosis Using Convolutional Neural Networks. Proceedings of the 2024 IEEE 7th International Conference on Advanced Technologies, Signal and Image Processing (ATSIP).

[28] Anders C.J., Neumann D., Samek W., Müller K.R., Lapuschkin S. (2021). Software for Dataset-wide XAI: From Local Explanations to Global Insights with Zennit, CoRelAy, and ViRelAy. arXiv.

[29] Böhle M., Eitel F., Weygandt M., Ritter K. (2019). Layer-Wise Relevance Propagation for Explaining Deep Neural Network Decisions in MRI-Based Alzheimer’s Disease Classification. Front. Aging Neurosci..

[30] Qu Y., Wang P., Liu B., Song C., Wang D., Yang H., Liu Y. (2021). AI4AD: Artificial intelligence analysis for Alzheimer’s disease classification based on a multisite DTI database. Brain Disord..

[31] Rehman S.U., Tarek N., Magdy C., Kamel M., Abdelhalim M., Melek A., Mahmoud L.N., Sadek I. (2024). AI-based tool for early detection of Alzheimer’s disease. Heliyon.

[32] Bloch L., Friedrich C.M. (2022). Machine Learning Workflow to Explain Black-box Models for Early Alzheimer’s Disease Classification Evaluated for Multiple Datasets. SN Comput. Sci..

[33] Alarjani M. Alzheimer’s Disease Detection based on Brain Signals using Computational Modeling. Proceedings of the 2024 Seventh International Women in Data Science Conference at Prince Sultan University (WiDS PSU).

[34] Bogdanovic B., Eftimov T., Simjanoska M., Bogdanovic B., Eftimov T., Simjanoska M. (2022). In-depth insights into Alzheimer’s disease by using explainable machine learning approach. Sci. Rep..

[35] Guan H., Wang C., Cheng J., Jing J., Liu T. (2022). A parallel attention--augmented bilinear network for early magnetic resonance imaging--based diagnosis of Alzheimer’s disease. Hum. Brain Mapp..

[36] Yousefzadeh N., Tran C., Ramirez-Zamora A., Chen J., Fang R., Thai M.T. (2024). Neuron-level explainable AI for Alzheimer’s Disease assessment from fundus images. Sci. Rep..

[37] Al Alatrany A.S., Khan W., Hussain A., Kolivand H., Al-Jumeily D. (2024). An explainable machine learning approach for Alzheimer’s disease classification. Sci. Rep..

[38] Umeda-Kameyama Y., Kameyama M., Tanaka T., Son B.-K., Kojima T., Fukasawa M., Iizuka T., Ogawa S., Iijima K., Akishita M. (2021). Screening of Alzheimer’s disease by facial complexion using artificial intelligence. Aging.

[39] KAchilleos G., Leandrou S., Prentzas N., Kyriacou P.A., Kakas A.C., Pattichis C.S. Extracting Explainable Assessments of Alzheimer’s disease via Machine Learning on brain MRI imaging data. Proceedings of the 2020 IEEE 20th International Conference on Bioinformatics and Bioengineering (BIBE).

[40] Alvarado M., Gómez D., Nuñez A., Robles A., Marecos H., Ticona W. Implementation of an Early Detection System for Neurodegenerative Diseases Through the use of Artificial Intelligence. Proceedings of the 2023 IEEE XXX International Conference on Electronics, Electrical Engineering and Computing (INTERCON).

[41] Liu Y., Tang L., Liao C., Zhang C., Guo Y., Xia Y., Zhang Y., Yao S. (2023). Optimized Dropkey-Based Grad-CAM: Toward Accurate Image Feature Localization. Sensors.

[42] Ang C.K.E., Zhao X., Acharya U.R., Cheong K.H. (2021). Application of Artificial Intelligence techniques for the detection of Alzheimer’s disease using structural MRI images. Biocybern. Biomed. Eng..

[43] Yao Z., Wang H., Yan W., Wang Z., Zhang W., Wang Z., Zhang G. (2023). Artificial intelligence-based diagnosis of Alzheimer’s disease with brain MRI images. Eur. J. Radiol..

[44] Yi F., Yang H., Chen D., Qin Y., Han H., Cui J., Bai W., Ma Y., Zhang R., Yu H. (2023). XGBoost-SHAP-based interpretable diagnostic framework for alzheimer’s disease. BMC Med. Inform. Decis. Mak..

[45] Kothari N., Syed M.R., Joshi Y., Gawade A. (2023). EADDA: Towards Novel and Explainable Deep Learning for Early Alzheimer’s Disease Diagnosis Using Autoencoders. Int. J. Intell. Syst. Appl. Eng..

[46] Shukla A., Upadhyay S., Bachan P.R., Bera U.N., Kshirsagar R., Nathani N. Dynamic Explainability in AI for Neurological Disorders: An Adaptive Model for Transparent Decision-Making in Alzheimer’s Disease Diagnosis. Proceedings of the 2024 IEEE 13th International Conference on Communication Systems and Network Technologies (CSNT).

[47] Al-Bakri F.H., Bejuri W.M.Y.W., Al-Andoli M.N., Ikram R.R.R., Khor H.M., Sholva Y., Ariffin U.K., Yaacob N.M., Abas Z.A., Abidin Z.Z. (2025). A Feature-Augmented Explainable Artificial Intelligence Model for Diagnosing Alzheimer’s Disease from Multimodal Clinical and Neuroimaging Data. Diagnostics.

[48] Zhang Y., Xu F., Zou J., Petrosian O.L., Krinkin K.V. XAI Evaluation: Evaluating Black-Box Model Explanations for Prediction. Proceedings of the 2021 II International Conference on Neural Networks and Neurotechnologies (NeuroNT).

[49] Covert I., Lundberg S., Lee S.I. (2020). Feature Removal Is a Unifying Principle for Model Explanation Methods. arXiv.

[50] Selvaraju R.R., Cogswell M., Das A., Vedantam R., Parikh D., Batra D. (2016). Grad-CAM: Visual Explanations from Deep Networks via Gradient-based Localization. arXiv.

[51] Saif F.H., Al-Andoli M.N., Bejuri W.M.Y.W., Saif F.H., Al-Andoli M.N., Bejuri W.M.Y.W. (2024). Explainable AI for Alzheimer Detection: A Review of Current Methods and Applications. Appl. Sci..

