# Correction: Al-bakri et al. A Hybrid Explainable AI Framework (HXAI) for Accurate and Interpretable Diagnosis of Alzheimer’s Disease. *Diagnostics* 2025, *15*, 3118

**DOI:** 10.3390/diagnostics16071031

**Published:** 2026-03-30

**Authors:** Fatima Hasan Al-bakri, Wan Mohd Yaakob Wan Bejuri, Mohamed Nasser Al-Andoli, Raja Rina Raja Ikram, Hui Min Khor, Mohd Syafiq Mispan, Norhazwani Md Yunos, Noor Fazilla Abd Yusof, Muhammad Hafidz Fazli Md Fauadi, Abdul Syukor Mohamad Jaya, Nor Aiza Moketar, Noorrezam Yusop, Kharismi Burhanudin, Tyanita Puti Marindah, Anugrayani Bustamin, Zahir Zainuddin, Deasy Wahyuni, Umi Kalsom Ariffin

**Affiliations:** 1 Faculty of Information and Communication Technology, Universiti Teknikal Malaysia Melaka, Melaka 76100, Malaysia; forignn@gmail.com (F.H.A.-b.); raja.rina@utem.edu.my (R.R.R.I.); nor.aiza@utem.edu.my (N.A.M.); noorrezam@utem.edu.my (N.Y.); 2 Faculty of Artificial Intelligence and Cyber Security, Universiti Teknikal Malaysia Melaka, Melaka 76100, Malaysia; wanie@utem.edu.my (N.M.Y.); elle@utem.edu.my (N.F.A.Y.); hafidz@utem.edu.my (M.H.F.M.F.); syukor@utem.edu.my (A.S.M.J.); kharismi@utem.edu.my (K.B.); 3 Faculty of Computing Informatics, Multimedia University, Cyberjaya 63100, Malaysia; 4 Department of Medicine, Faculty of Medicine, University of Malaya, Kuala Lumpur 50603, Malaysia; hmkhor@um.edu.my; 5 Fakulti Teknologi dan Kejuruteraan Elektronik dan Komputer, Universiti Teknikal Malaysia Melaka, Melaka 76100, Malaysia; syafiq.mispan@utem.edu.my; 6 Faculty of Engineering, Universitas Hasanuddin, Gowa 92171, Indonesia; tyanitaputi@unhas.ac.id (T.P.M.); anugrayani@unhas.ac.id (A.B.); zahir@unhas.ac.id (Z.Z.); 7 Fakultas Ilmu Komputer, Universitas Dumai, Riau 28811, Indonesia; deasywahyuni1@gmail.com; 8 Lee Kong Chian Faculty of Engineering and Science, Universiti Tunku Abdul Rahman, Kajang 43000, Malaysia; madamumikalsom2025@gmail.com

**Removal of an Author**

The Alzheimer’s Disease Neuroimaging Initiative was included as an author in the original publication [1]. The corrected Author Contributions and Acknowledgements Sections appear here. The authors state that the scientific conclusions are unaffected. This correction was approved by the Academic Editors. The original publication has also been updated.

**Author Contributions**

Conceptualization, F.H.A.-b.; Methodology, F.H.A.-b. and W.M.Y.W.B.; Validation, W.M.Y.W.B., F.H.A.-b., R.R.R.I., and H.M.K.; Formal analysis, F.H.A.-b.; Investigation, F.H.A.-b., W.M.Y.W.B., M.S.M., N.M.Y., N.F.A.Y., M.H.F.M.F., A.S.M.J., N.A.M., N.Y., K.B., T.P.M., A.B., Z.Z., D.W., and U.K.A.; Writing—original draft preparation, F.H.A.-b.; Writing—review and editing, all authors; Visualization, F.H.A.-b.; Supervision, W.M.Y.W.B.; Guidance on ensemble learning and XAI, M.N.A.-A.; Medical validation and clinical review, H.M.K. All authors have read and agreed to the published version of the manuscript.

**Acknowledgements**

The authors would like to thank the Optimas Research Group, Center for Advanced Computing Technology (C-ACT), Fakulti Kecerdasan Buatan dan Keselamatan Siber (FAIX), Fakulti Teknologi Maklumat dan Komunikasi (FTMK), and Centre for Research and Innovation Management (CRIM), Universiti Teknikal Malaysia Melaka (UTeM), for providing the facilities and support for this research. The authors also thank the Research Management Center (RMC), Multimedia University, for their valuable support in this research. Data collection and sharing for this project was funded by the Alzheimer’s Disease Neuroimaging Initiative (ADNI) (National Institutes of Health Grant U01 AG024904) and DOD ADNI (Department of Defense award number W81XWH-12-2-0012). ADNI investigators contributed to the design and implementation of ADNI and/or provided data but did not participate in the analysis or writing of this report.
